# College Opening

**Published:** 1899-09

**Authors:** 


### College Opening.

The beginning of the academic year of nearly all educational institutions is near at hand. The teachers and pupils will be soon gathering in obedience to the demands of the people for education.

This requisite is everywhere present. Our attention is more particularly directed to the dental colleges of our country. These will doubtless have for the coming year a larger attendance than ever before. The attention was never so generally nor so favorably given to the importance and value of correct dental practice as now. It was never more favorably regarded as a life vocation by those seeking a professional calling. This accounts in a large degree for the large increasing number of applications for entrance to the dental colleges.

The indications are that there will be a greater number of applications than ever before. This occurs because of more thorough work being now done by our colleges than ever before. Students are in the main better prepared for their work, and the public appreciating the services of the dentist more, there is a greater demand. Much has been said about the excessive number entering the profession. This in a degree applies to the incompetents; but there is far from an excess of thoroughly prepared dentists. The profession is, however, crowded with those deficient in professional ability. The fact of the growing demand for a better class of dentists ought to be an incentive to those seeking to enter and to more thoroughly prepare themselves, and to put forth their utmost effort for this purpose.

This condition of things ought to be a great stimulus to the colleges to make their work more thorough, and, indeed, ought to be an inducement to them to eliminate from their classes those who are unprepared for taking up the course. More care should certainly be exercised by many, if not all, our colleges in this respect.

